# Rodent Area Prostriata Converges Multimodal Hierarchical Inputs and Projects to the Structures Important for Visuomotor Behaviors

**DOI:** 10.3389/fnins.2021.772016

**Published:** 2021-11-02

**Authors:** Chang-Hui Chen, Jin-Meng Hu, Shun-Yu Zhang, Xiao-Jun Xiang, Sheng-Qiang Chen, Song-Lin Ding

**Affiliations:** ^1^Key Laboratory of Neuroscience, School of Basic Medical Sciences, Institute of Neuroscience, The Second Affiliated Hospital, Guangzhou Medical University, Guangzhou, China; ^2^Allen Institute for Brain Science, Seattle, WA, United States

**Keywords:** pretectal region, lateral geniculate nucleus, pulvinar, anterior thalamic nucleus, zona incerta, connectivity, limbic cortex, subcortical visual pathways

## Abstract

Area prostriata is a limbic structure critical to fast processing of moving stimuli in far peripheral visual field. Neural substrates underlying this function remain to be discovered. Using both retrograde and anterograde tracing methods, the present study reveals that the prostriata in rat and mouse receives inputs from multimodal hierarchical cortical areas such as primary, secondary, and association visual and auditory cortices and subcortical regions such as the anterior and midline thalamic nuclei and claustrum. Surprisingly, the prostriata also receives strong afferents directly from the rostral part of the dorsal lateral geniculate nucleus. This shortcut pathway probably serves as one of the shortest circuits for fast processing of the peripheral vision and unconscious blindsight since it bypasses the primary visual cortex. The outputs of the prostriata mainly target the presubiculum (including postsubiculum), pulvinar, ventral lateral geniculate nucleus, lateral dorsal thalamic nucleus, and zona incerta as well as the pontine and pretectal nuclei, most of which are heavily involved in subcortical visuomotor functions. Taken together, these results suggest that the prostriata is poised to quickly receive and analyze peripheral visual and other related information and timely initiates and modulates adaptive visuomotor behaviors, particularly in response to unexpected quickly looming threats.

## Introduction

Area prostriata (prostriata, Pro) is a limbic cortical region located at the junction of the retrosplenial cortex (RS), caudal presubiculum (PrS), caudal parasubiculum (PaS), and medial visual cortex in human and non-human primates (Sanides, 1969; Allman and Kaas, 1971; Sousa et al., 1991; Morecraft et al., 2000; Ding et al., 2003; Rockland, 2012). The prostriata has recently been found to play important roles in fast processing of information from far peripheral visual field (particularly for fast-moving objects) in both human and non-human primates (Yu et al., 2012; Mikellidou et al., 2017; Tamietto and Leopold, 2018). In addition, peripheral visual hypersensitivity might be associated with panic disorders and agoraphobia (Caldirola et al., 2011). Currently, the mechanisms and neural circuits underlying the fast processing of peripheral visual information remain largely unknown, although subcortical processing pathways were proposed and more intensely studied (Conrad and Stumpf, 1975; McFadyen, 2019; Isa et al., 2021).

Cortical processing of visual information is generally thought to be split into dorsal (where) and ventral (what) streams. Starting from V1, each stream processes visual information via hierarchical extrastriate regions before reaching to the frontal premotor or temporal limbic cortices, and the latter two regions then initiate and modulate adaptive or defensive responses to specific visual stimuli (Mesulam, 1998; Angelucci and Rosa, 2015). To enable fast processing of peripheral vision, shorter visual pathways should exist in the brain to avoid multiple relays before reaching the limbic cortices such as the prostriata. Unfortunately, the lack of data on the afferent projections of the prostriata has hindered the exploration of possible shorter pathways in human and non-human primates likely due to difficult access to the prostriata, which is located deep into the anterior calcarine fissure, and thus difficult to be targeted with neural tracer injections. Scattered information about the efferent projections of the prostriata is available in monkey via retrograde tracer injections into some cortical regions. Specifically, retrogradely labeled neurons were observed in the prostriata after the tracer injections into the primary visual cortex (V1; Sousa et al., 1991), the middle temporal visual area (Rosa et al., 1993; Palmer and Rosa, 2006), the orbitofrontal cortex (Barbas, 1993; Cavada et al., 2000), the rostral cingulate motor cortex (Morecraft et al., 2000), the auditory association cortex (Falchier et al., 2010), the parietal area PGm (Passarelli et al., 2018), the dorsal prefrontal cortex (area 8b) (Reser et al., 2013; Bakola et al., 2021), and the frontal pole (Burman et al., 2011).

The discovery of the rodent equivalent of primate prostriata (Ding, 2013; Lu et al., 2020) enables systematic investigation of brain-wide connectivity of the prostriata via direct retrograde and anterograde tracer injections into the prostriata. This discovery has allowed a brain-wide survey on the afferent projections to mouse prostriata using an available large connectivity dataset (Hu et al., 2020). This recent study reveals that mouse prostriata receives inputs from association visual and auditory cortices (V2 and A2, respectively), from many limbic structures [e.g., ectorhinal cortex (ECT), postrhinal cortex (PoR), medial entorhinal cortex (MEC), RS, PrS, and subiculum (Sub)] and from subcortical structures [e.g., anterior thalamic nuclei (ATN) and claustrum (Cla)]. A more interesting finding is that the prostriata receives direct projections from V1 and primary auditory cortex (A1) (Ding et al., 2003; Lu et al., 2020). These direct primary sensory inputs appear to be unique to the prostriata since they were not reported for other limbic cortices. ATN projections to the prostriata were also reported in tree shrew (Conrad and Stumpf, 1975). Recently, direct projections from the dorsal lateral geniculate nucleus (DLG) to the prostriata in human brain have been suggested based on indirect tracing methods (tractography from human diffusion dataset) (Kurzawski et al., 2020). This pathway, if confirmed using direct tracing methods, would represent the shortest one from the retina to the prostriata since only one relay (DLG) exists between the two. As for the efferent projections of the prostriata in rodents, two major target regions have recently been revealed: V1 and contralateral prostriata (Chen et al., 2020; Lu et al., 2020). However, brain-wide efferent projections of the prostriata have not been investigated systematically with tracer injections directly placed in this area.

Therefore, the first aim of this study is to systematically examine the sources of afferent projections to the prostriata in rat and compare them with mouse (Hu et al., 2020). The second aim is to reveal brain-wide efferent projections from the prostriata of the rat and mouse to gain insights about their impact on downstream structures. The third aim is to investigate the possibility of direct projections from the DLG to the prostriata using direct pathway tracing methods.

## Materials and Methods

### Animals

The experiments in this study were carried out on 42 adult Sprague–Dawley rats of both sexes weighing 280–310 g (Beijing Vital River Laboratory Animal Technology Co., Ltd., Beijing, China). Some of the animals were the same as those used in our previous study (Chen et al., 2020). All animals were housed in the same room with a suitable illumination period and fixed room temperature, as well as free access to food and water. All operations in this study were performed under deep anesthesia to alleviate their suffering. All experimental procedures were followed in accordance with the protocols that have been approved by the Institutional Animal Care and Use Committee.

### Surgery Procedure and Tracer Injections

The specific surgical procedure was described in our recent study (Chen et al., 2020). Briefly, after deep anesthesia with sodium pentobarbital (40 mg/kg, i.p.), the rats were fixed in a stereotaxic frame, and then one 2-cm midline incision was made on the top of the cranial skin. Next, the height of the nose clip was adjusted to make the bregma and lambda at the same level, and a small blur hole of appropriate size was made on the skull overlying the target brain regions in line with the coordinates. The target regions include the prostriata and DLG [for both biotinylated dextran amine (BDA) and Fluoro-Gold (FG) injections] as well as the RS, lateroposterior nucleus-pulvinar complex (LP-Pul), ventral lateral geniculate nucleus (VLG), and pretectal nucleus (PTN) (for FG injections). All the stereotaxic coordinates used in this study were derived from the rat brain atlas of Paxinos and Watson (2013). Next, 0.1 μl of 10% BDA (10,000 MW, Thermo Fisher Scientific, Waltham, MA, United States) or 4% FG (Fluorochrome Inc., Denver, CO, United States) was pressure injected into the target brain regions of one hemisphere using a 0.5-μl Hamilton syringe. The needle was held in place for 10 min before being slowly pulled out, and then the incision was sutured. Finally, after waking up in a warm bed, the rats were returned to their home cage, where they were free to get water and food.

### Tissue Processing

After 7–10 days of survival, the rats were anesthetized with sodium pentobarbital (40 mg/kg, i.p.) and perfused transcardially with 0.9% saline followed by 4% paraformaldehyde (PFA) in chilled 0.1 M of phosphate buffer (PB; pH 7.3). The brains were extracted and postfixed in 4% PFA at 4°C overnight, and then cryoprotected in 0.1 M of PB containing 15 and 30% sucrose successively for 3–4 days. The brains were divided into two hemispheres with a cut along the midline and the hemisphere with a tracer injection cut into sequential sagittal sections of 40 μm in thickness using a freezing microtome. Sections from the cases with BDA injections were visualized by mean of the histochemistry for BDA tracing (see below), while those with FG injections were observed directly under an epifluorescent microscope (Leica Microsystems GmbH, Wetzlar, Germany; DM6B) or processed for immunohistochemistry (IHC) with anti-FG antibody according to the following IHC procedure.

### Immunohistochemistry for Calbindin-D28k and Fluoro-Gold

The IHC for Calbindin-D28k (CB) and FG was carried out in accordance with the standard procedure to facilitate the identification of the prostriata (see Lu et al., 2020) or turn the fluorescent FG into non-fluorescent products (see Chen et al., 2020). Briefly, after rinses in 0.1 M of PB, the sections were incubated in 3% hydrogen peroxide solution for 10 min and then in 5% bovine serum albumin (BSA) for 40 min for blocking. Next, sections were incubated at 4°C overnight with a solution containing 0.3% triton X-100 and the primary antibody [mouse anti-CB (66394-1-Ig, 1:1,000, ProteinTech Group, Inc., Chicago, IL, United States) or rabbit anti-FG (AB153-I, 1:10,000, Sigma-Aldrich, St. Louis, MO, United States)]. Then, the sections were incubated with the secondary antibody solution (biotinylated goat anti-mouse/rabbit IgG, Boster Biological Technology, Pleasanton, CA, United States) followed by the Streptavidin-Biotin Complex solution (SABC kit, Boster Biological Technology) for 60 min each. After rinses, the sections were visualized by incubating in 0.1 M of PB containing 0.05% 3,3′-diaminobenzidine (DAB) and 0.01% hydrogen peroxide. Finally, the sections were mounted on chrome alum and gelatin-coated slides, dehydrated in gradient alcohol and xylene, and coverslipped.

### Histochemistry for Biotinylated Dextran Amine Tracing

The procedure for BDA histochemistry was described in our previous study (Chen et al., 2020). Briefly, after rinses with 0.1 M of PB, the sections from the cases with BDA injections were incubated in 0.3% Triton X-100 in 0.1 M of PB for 60 min and in Streptavidin-Biotin Complex solution (SABC kit, Boster Biological Technology) for 120 min at room temperature in sequence. After rinses in 0.1 M of PB, the sections were visualized with 0.1 M of PB containing 0.05% DAB and 0.01% hydrogen peroxide. Then the sections were mounted on chrome alum and gelatin-coated slides, dehydrated in gradient alcohol and xylene, and finally coverslipped.

### Image Acquisition and Processing

Sections stained with the histochemistry and IHC were digitized using a histological section scanner (Aperio CS2, Leica). For a few cases with FG injections in the prostriata, retrogradely labeled neurons and their brain-wide distribution were searched and photographed directly under an epifluorescent microscope (Leica DM6B). For the cases with non-Pro injections (e.g., FG injections in the VLG and PTN), selected sections were also photographed directly under the epifluorescent microscope for evaluation of the injection sites and laminar locations of the labeled neurons in the prostriata. All the captured images are finally processed in Photoshop 2020 for image clipping, brightness adjustment, and image typesetting.

## Results

### Localization of Area Prostriata

**FIGURE 1:**
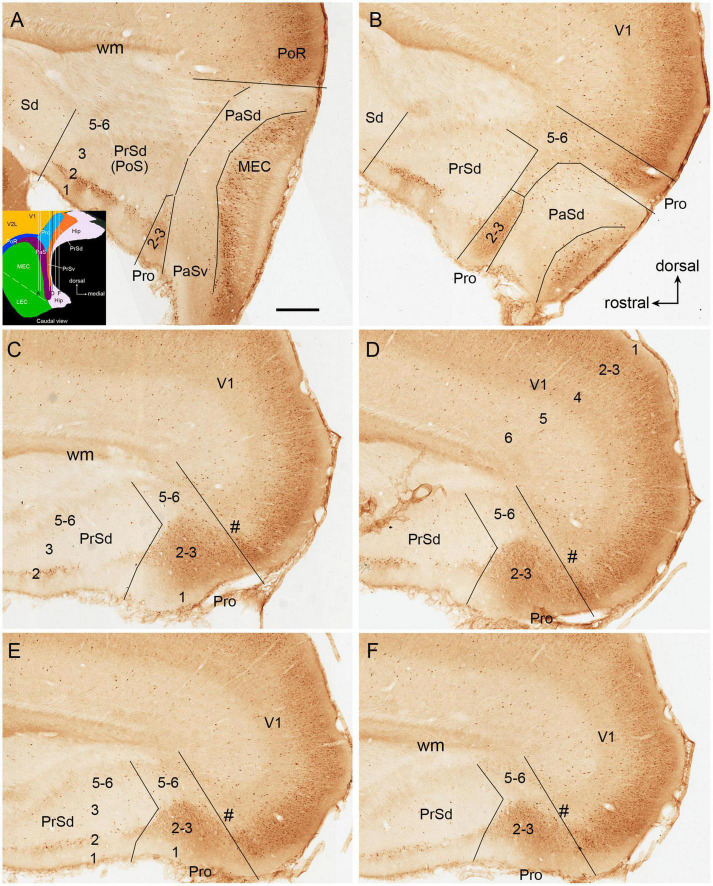
Calbindin-D28k (CB) immunostaining in rat prostriata and adjoining regions. **(A–F)** Sequential sagittal sections from lateral **(A)** to medial **(F)** levels. The dorsal and rostral orientations are shown in **(B)** for all sagittal sections. The inset in **(A)** shows a schematic map of the region containing the prostriata (Pro) and adjoining areas (from caudal view) as well as the orientation of the sections shown in **(A–F)** (indicated by the vertical lines). Arabic numbers (1–6) indicate cortical layers. **(A,B)** Two sections at the lateral levels showing a small portion of layers 2–3 of the prostriata located between PrSd and parasubiculum (PaS). Note the overall faint CB labeling in the PaS. **(C–F)** Four sections at progressively more medial levels showing typical location of the prostriata. Most parts of the prostriata adjoin PrSd rostrally and the medial visual cortex (mainly V1) dorsocaudally. Note the narrow zone (marked by #) with weaker labeling at the junction between the prostriata and visual cortex. At all levels **(A–F)**, strong and weak CB labeling exists in layers 2–3 and 5–6 of the prostriata, respectively. Stronger CB labeling in layer 2 and weaker labeling in other layers of the dorsal presubiculum [PrSd; i.e., postsubiculum (PoS)] is also obvious. Sd, dorsal subiculum; PoR, postrhinal cortex; MEC, medial entorhinal cortex; V1, primary visual cortex; Hip, hippocampus; wm, white matter. Bar: 500 μm in **(A)** for all panels.

To facilitate identification of the prostriata in the rat, sequential sagittal sections were stained with CB. As described previously (Lu et al., 2020), CB strongly and weakly labels layers 2–3 and 5–6 of the prostriata, respectively (Figure 1). In contrast, the regions located anterior to the prostriata (i.e., PrSd-PoS) or ventral to the prostriata (i.e., PaS) display overall weaker labeling. Specifically, PrSd shows relatively strong labeling in layer 2 with weak labeling in other layers (Figures 1A–F), while PaS contains scattered CB-positive neurons (Figures 1A,B). Dorso-posteriorly, the prostriata mainly adjoins medial visual cortex (V1 and medial V2), where strong CB labeling is also seen in layers 2–3. However, there is a narrow zone with weaker labeling at the junction between the prostriata and visual cortex on most of the sagittal sections (Figures 1C–F). In addition, at the lateral levels of the prostriata (where PaS appears), narrower prostriata still exists dorsal to PaS (Figure 1B), while a small portion of layers 2–3 of the prostriata is located between PrSd and PaS (Figures 1A,B). The location of mouse prostriata is similar to that of the rat, and the markers for mouse prostriata have been detailed in our recent articles (Chen et al., 2020; Hu et al., 2020; Lu et al., 2020). The boundaries of the prostriata with RS subdivisions and medial V2 can be clearly identified using gene markers such as *Igfbp5*, *C1ql2*, and *Rorb*, as demonstrated in our recent study (see Figures 1, 3, 11 of Lu et al., 2020). In macaque monkey (Ding et al., 2003) and marmoset (Paxinos et al., 2012), relatively strong CB expression (compared with the RS) was also shown in the superficial layers of the prostriata.

### Injection Sites of the Tracers Fluoro-Gold and Biotinylated Dextran Amine

The tracer injections targeting the prostriata often involved overlying V1 and adjoining PrSd. Based on the location and extent of the prostriata revealed with CB staining (Figure 1), the injection sites of FG and BDA involved in rat prostriata were divided into four groups: the prostriata plus all layers of the overlying V1 (Pro + V1; 10 cases), prostriata plus layer 6 of the overlying V1 (Pro + V1L6, eight cases), prostriata plus adjoining PrSd (Pro + PrSd; two cases), and prostriata only (Pro; five cases). Additional five cases with injections restricted in V1 were used as control. The results described below are mainly based on the cases with injections in the prostriata only and Pro + V1L6, although in other groups, similar results about the afferent and efferent projections of the prostriata were confirmed. To reveal the origins of cells projecting from the prostriata to its main targets, additional FG injections were placed in four of the target regions including the LP-Pul, RS, VLG, and PTN (three to five cases each). To examine the connections of the DLG with V1 and the prostriata, five additional injections of FG or BDA were placed in the DLG (two to three cases each).

### Brain-Wide Afferent Projections to Area Prostriata in Rat

**FIGURE 2:**
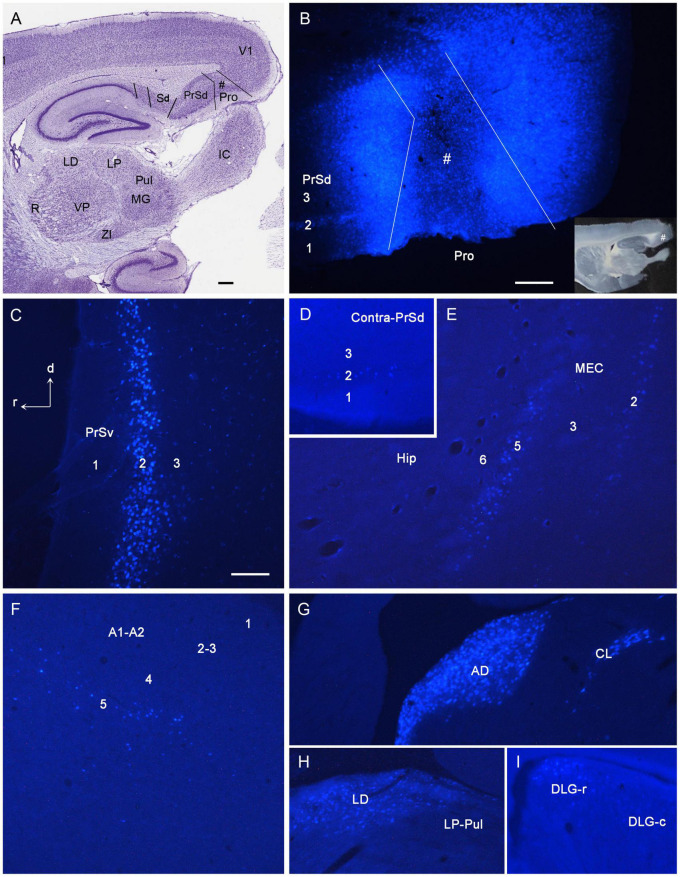
Examples of retrogradely labeled neurons resulted from a Fluoro-Gold (FG) injection in rat prostriata. All panels are from sagittal sections. The dorsal [d] and rostral [r] orientation is shown in **(C)** for all panels. **(A)** A Nissl-stained section at the level of the injection (#) showing the locations of the prostriata (Pro) and nearby regions. **(B)** A fluorescent image showing the FG injection site (#) restricted to the prostriata. The inset **(B)** shows the same unprocessed section containing the injection sites. Note the retrogradely labeled neurons in layer 2 of PrSd. **(C)** FG retrogradely labeled neurons in layer 2 of PrSv. **(D)** FG-labeled neurons in layer 2 of contralateral PrSd. **(E)** FG-labeled neurons in layers 2 and 5 of medial entorhinal cortex (MEC). **(F)** FG-labeled neurons in layer 5 of the auditory cortex (both primary and secondary cortices, A1–A2). **(G)** FG-labeled neurons in anterodorsal (AD) and centrolateral (CL) nuclei of the thalamus. **(H)** FG-labeled neurons in laterodorsal thalamic nucleus (LD). Note the absence of labeled neurons in the lateroposterior nucleus–pulvinar complex (LP-Pul). **(I)** FG-labeled neurons in the rostral part of DLG (DLG-r). R, reticular thalamic nucleus; VP, ventroposterior thalamic nucleus; MG, medial geniculate nucleus; ZI, zona incerta; IC, inferior colliculus. Bars: 370 μm **(A)**; 100 μm **(C)**; 250 μm **(B)** for **(B,D–I)**.

**FIGURE 3:**
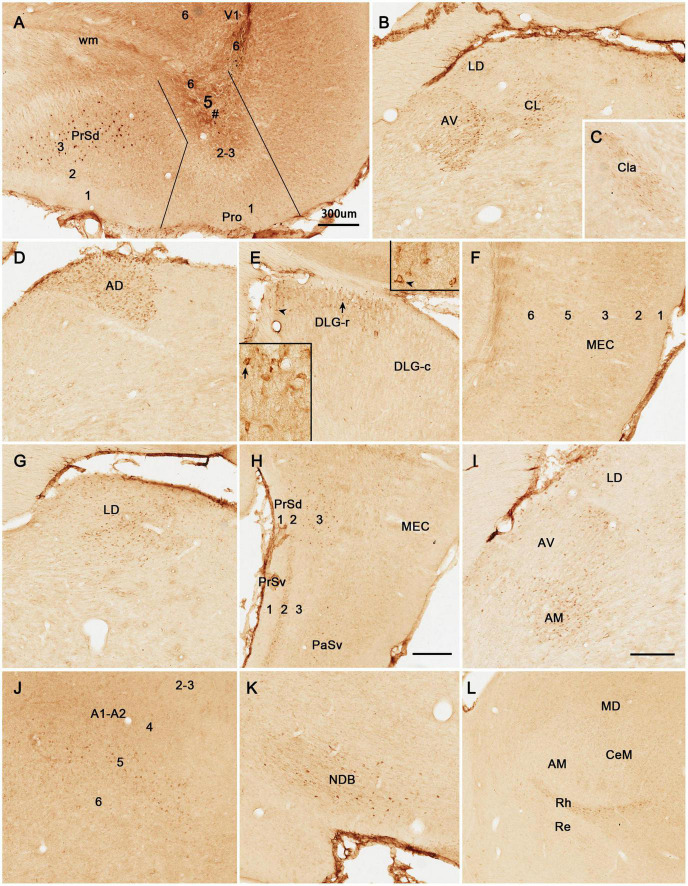
Examples of retrogradely labeled neurons resulted from another Fluoro-Gold (FG) injection in rat prostriata. All panels are from sagittal sections. In this case, FG was turned into non-fluorescent products via immunohistochemistry with anti-FG antibody. The injection (#) is mainly located in the prostriata (Pro) with involvement in layer 6 of overlying V1 **(A)**. Retrogradely labeled neurons are shown in layers 2–3 of PrSd **(A,H)**, anteroventral thalamic nucleus (AV) and CL **(B)**, claustrum (Cla in **C**), AD **(D)**, DLG-r **(E)**, layers 2 and 5 of medial entorhinal cortex (MEC) **(F)**, LD **(G)**, AM **(I)**, layer 5 of A1–A2 **(J)**, nucleus of diagonal band (NDB in **K**), and rhomboid nucleus (Rh in **L**). Note the two insets in **(E)** showing high power views of the labeled neurons indicated by arrow and arrowhead, respectively. Bars: 300 μm **(A)** for **(A–G,J–L)**; 350 μm **(H)**; 250 μm **(I)**.

Following FG injections in the prostriata only (e.g., Figures 2A,B) or Pro + V1L6 (e.g., Figure 3A), retrogradely labeled neurons are mainly found in layers 2–3 of PrS (Figures 2B,C, 3A), layers 2 and 5 of the MEC (Figures 2E, 3F,H), and layer 5 of A1–A2 (Figures 2F, 3J), V1–V2, ECT, PoR, RS, and pyramidal cell layer of Sub (not shown) with fewer in layers 2–3 of lateral entorhinal cortex (LEC), piriform cortex (Pir), and medial orbitofrontal cortex (ORBm) (not shown). In the thalamus, labeled neurons are mainly observed in the anterodorsal (AD), anteroventral (AV), anteromedial (AM), and laterodorsal (LD) nuclei (Figures 2G,H, 3B,D,G,I) as well as in the parataenial (Pt), centrolateral (CL), and rhomboid (Rh) nuclei (e.g., Figures 2G, 3B,L). Interestingly, some labeled neurons are also seen in the rostral part of the DLG (DLG-r), and this is true for cases with FG injections in both the prostriata only and Pro + V1L6 (e.g., Figures 2I, 3E). In cases with FG injections in Pro + V1 (all layers), however, much more labeled neurons were seen in the DLG-r (see “Direct Projections From DLG to Prostriata” section). This is consistent with the well-known finding in rodent that the DLG (including DLG-r) predominantly innervates layer 4 and deep layer 3 of V1 but not layers 5 and 6 of V1. In other subcortical regions, labeled neurons are also detected in the Cla (Figure 3C), nucleus of diagonal band (NDB; Figure 3K), and locus coeruleus (LC) with a few neurons in the medial septal nucleus (MSN; not shown). Contralaterally, many retrogradely labeled neurons are observed in layer 2 of PrSd (e.g., Figure 2D) and layers 2–3 of the prostriata (not shown, but see Chen et al., 2020).

**FIGURE 4:**
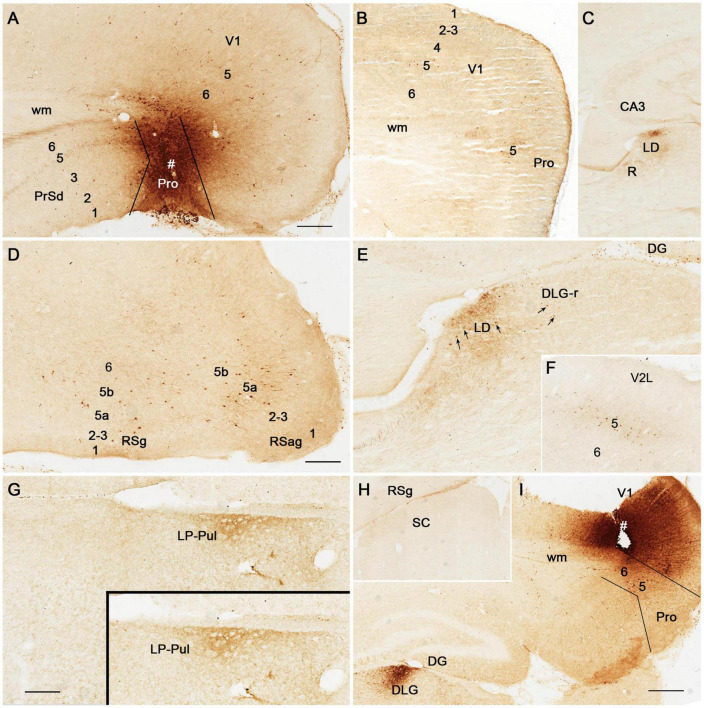
Retrograde and anterograde tracing following a biotinylated dextran amine (BDA) injection in rat prostriata. All panels are from sagittal sections. The BDA injection (#) is restricted to the prostriata (Pro in **A**). This injection resulted in both retrogradely labeled neurons and anterogradely labeled axon terminals. Labeled neurons are found in layer 5 of V1 and Pro **(B)**, layer 5 (5a and 5b) of the granular (RSg) and agranular (RSag) retrosplenial cortices **(D)**, and LD and DLG-r **(E)** as well as in layer 5 of the lateral secondary visual cortex (V2L in **F**). Some labeled neurons in LD and DLG-r are indicated by the arrows in **(E)**. Labeled axon terminals are seen in layer 5 of PrSd and layer 6 of V1 **(A)**, LD and R **(C,E)**, layer 6 of RSg and RSag **(D)**, and the LP-Pul (**G**; with higher power view in the inset). Note that no retrogradely labeled neurons exist in LP-Pul. In the superior colliculus (SC), both labeled neurons and axon terminals are not detected **(H)**. As a control, following a BDA injection in the deep layers of V1 (# in **I**), dense terminal labeling is seen in the DLG **(I)**, while some labeled neurons are observed in layer 5 of the prostriata (Pro in **I**). In contrast, the BDA injection restricted in the prostriata **(A)** results in few terminal labeling in the DLG **(E)**. Bars: 300 μm **(A)** for **(A–C,F,H)**; 225 μm **(D)** for **(D,E)**; 200 μm **(G)**; 350 μm **(I)**.

**FIGURE 5:**
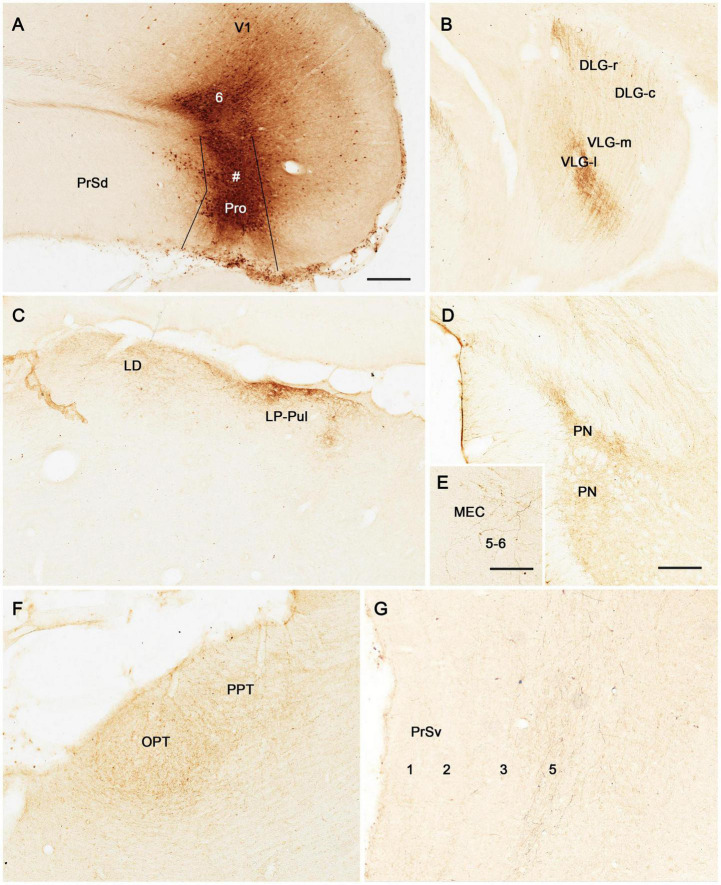
Anterograde tracing following a biotinylated dextran amine (BDA) injection in rat prostriata. All panels are from sagittal sections. The BDA injection is mainly located in the prostriata (# Pro) with involvement in layer 6 of overlying V1 **(A)**. This injection predominantly results in anterograde labeling of axon terminals **(B–G)**. Labeled axon terminals are mainly distributed in the DLG-r **(B)** and lateral part of ventral lateral geniculate nucleus (VLG-l; **B**), LD and LP-Pul **(C)**, lateral part of the pontine nucleus (PN in **D**), layers 5–6 of medial entorhinal cortex (MEC) **(E)**, olivary and posterior pretectal nuclei (OPT and PPT in **F**), and layer 5 of PrSv **(G)**. Bars: 300 μm **(A)** for **(A–C)**; 150 μm **(D)** for **(D,F)**; 100 μm in **(E)** for **(E,G)**.

BDA is a bidirectional tracer, usually revealing both retrogradely labeled neuronal somata and anterogradely labeled axon terminals and thus can be used as a retrograde tracer when neuronal somata are revealed. In two cases with BDA injections in the prostriata, both retrogradely labeled neurons and anterogradely labeled axon terminals are revealed (e.g., Figure 4), while in most cases, anterogradely labeled axon terminals are exclusively found (e.g., Figure 5). As shown in Figure 4, following a BDA injection restricted in the prostriata (see Figure 4A), retrogradely labeled neurons exist in layer 5 of V1, RS, and V2L (Figures 4A,B,D,F) as well as in the LD with fewer labeled neurons in DLG-r (Figure 4E, arrows) but none in the LP-Pul (Figure 4G).

### Brain-Wide Efferent Projections of Area Prostriata

#### Efferent Projections of Rat Prostriata

Following BDA injections in the prostriata only (e.g., Figure 4A) or Pro + V1L6 (e.g., Figure 5A), resulting axon terminal labeling is mainly seen in the LD (Figures 4C,E, 5C), LP-Pul (Figures 4G, 5C), lateral part of VLG (VLG-l; Figure 5B), lateral part of the pontine nucleus (PN; Figure 5D), olivary and posterior pretectal nuclei (OPT and PPT, respectively; Figure 5F), and layer 5 of PrS (Figure 5G). Sparse terminal labeling exists in layers 5–6 of the MEC (Figure 5E), layer 1 of the ORBm, and layers 5–6 of V1 and V2L (not shown), but none or few in the superior colliculus (SC; Figure 4H). Note that no and some labeled axon terminals are detected in the DLG-r in Figure 4E (from Pro injection) and Figure 5B (from Pro + V1L6 injection), respectively. This suggests that the terminal labeling in the DLG-r likely originates from layer 6 of V1. In contrast, after a BDA injection is restricted in the deep layers of V1, densely labeled axon terminals are detected in the DLG (Figure 4I). In addition, retrogradely labeled neurons are also found in layer 5 of the prostriata (Figure 4I), confirming our recent findings (Lu et al., 2020).

**FIGURE 6:**
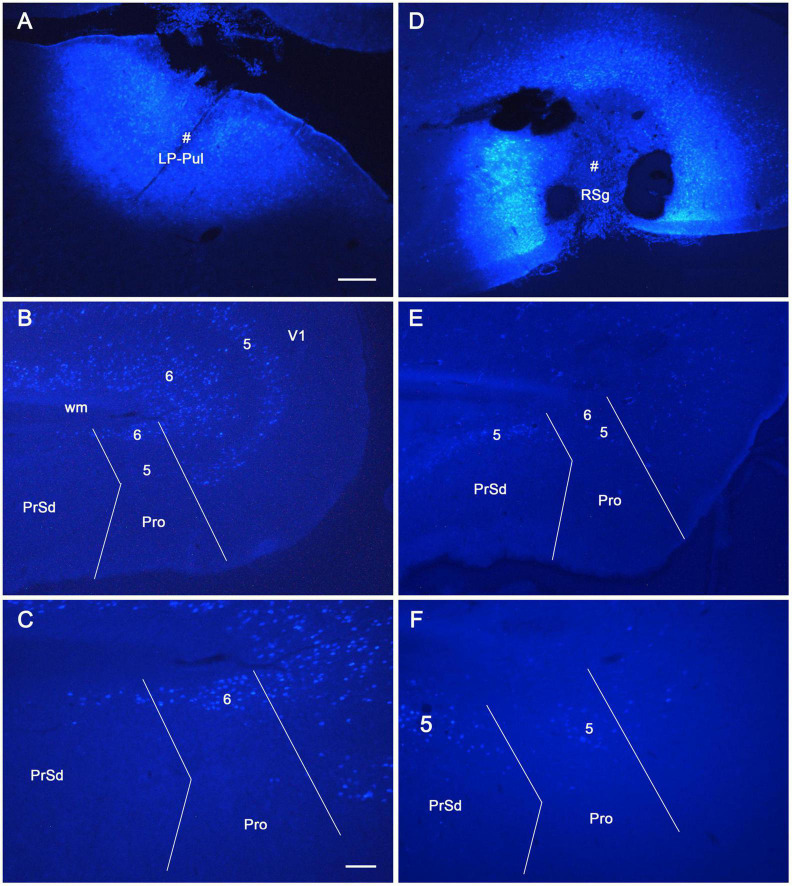
The origins of neurons projecting from rat prostriata to LP-Pul and RSg. All panels are from sagittal sections. **(A–C)** The Fluoro-Gold (FG) injection in LP-Pul (# in **A**) results in retrogradely labeled neurons mainly in layers 5–6 of the visual cortex (V1) and layer 6 of the prostriata (Pro in **B**). Many and a few of labeled neurons are detected in layers 6 and 5 of the prostriata, respectively, as shown in the higher power view of the prostriata **(C)**. Note that some labeled neurons are also seen in layer 6 of PrSd **(C)**. **(D–F)** Another FG injection in the RSg (# in **D**) results in some retrogradely labeled cells in layer 5 of the PrSd and prostriata **(E)** with higher power view in **(F)**. Bars: 250 μm **(A)** for **(A,B,D,E)**; 100 μm **(C)** for **(C,F)**.

**FIGURE 7:**
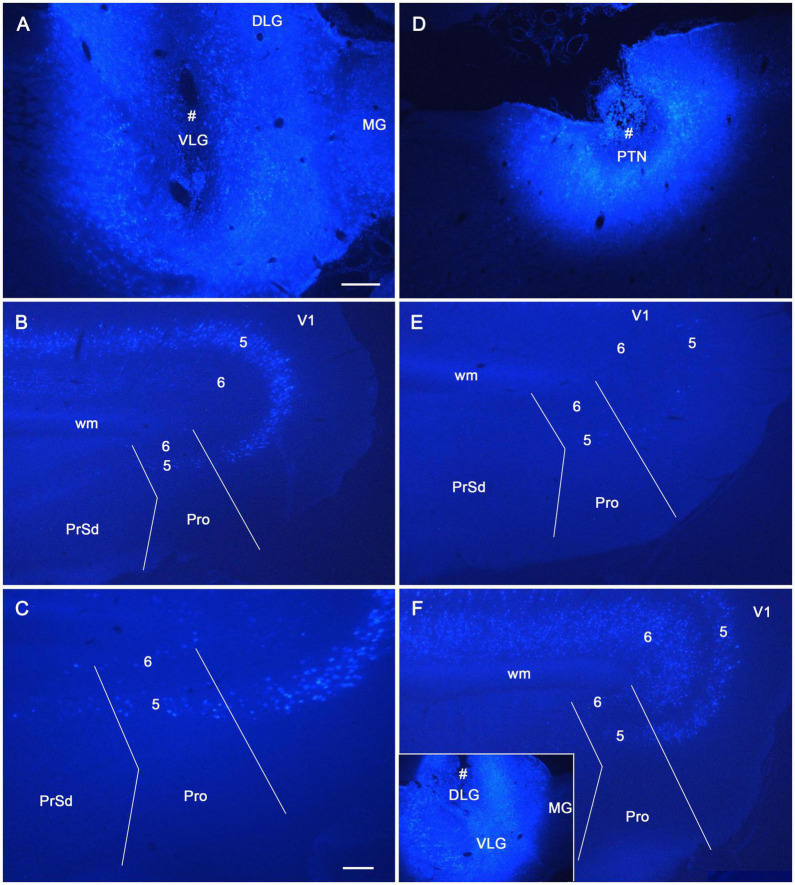
The origins of cells projecting from rat prostriata to VLG and PTN. All panels are from sagittal sections. **(A–C)** The Fluoro-Gold (FG) injection in the VLG (# in **A**) results in retrogradely labeled cells mainly in layer 5 of V1 and prostriata (**B**, with higher power view in **C**). Some labeled neurons are also found in layer 6 of the prostriata **(C)**. **(D,E)** Another FG injection in pretectal nucleus (PTN, # in **D**) results in some retrogradely labeled cells in layer 5 of V1 and prostriata **(E)**. **(F)** As a comparison, one FG injection located mostly in the DLG (# in the inset) produces many labeled neurons in layers 5–6 of V1 with much fewer in the prostriata. Note that the injection is only slightly involved in VLG. Bars: 250 μm **(A)** for **(A,B,D–F)**; 100 μm **(C)**.

To reveal the origins of neurons of efferent projections of the prostriata, FG injections were placed in four main target regions of the projections, LP-Pul, RSg, VLG and PTN. FG injections in the LP-Pul result in many and a few labeled neurons in layers 6 and 5 of the prostriata, respectively. Many labeled neurons are also seen in layers 5–6 of V1 (e.g., Figures 6A–C). RSg injections produce labeled neurons mainly in layer 5 of the prostriata and PrSd (e.g., Figures 6D–F). Following FG injections in the VLG, labeled neurons are mainly seen in layer 5 of the prostriata with much fewer in layer 6 (e.g., Figures 7A–C). Densely labeled neurons are also observed in layer 5 of V1 (Figure 7B). Finally, PTN injections lead to labeled neurons mostly in layer 5 of the prostriata and V1 with no labeling in other layers (e.g., Figures 7D,E). As a control for the VLG injections, DLG injections of FG result in many labeled neurons in both layers 5 and 6 of V1, with much fewer in layers 5 and 6 of the prostriata (e.g., Figure 7F). The sparse cell labeling in the prostriata likely originates from some involvement of the injection in the VLG (Figure 7F, inset).

#### Efferent Projections of Mouse Prostriata

**FIGURE 8:**
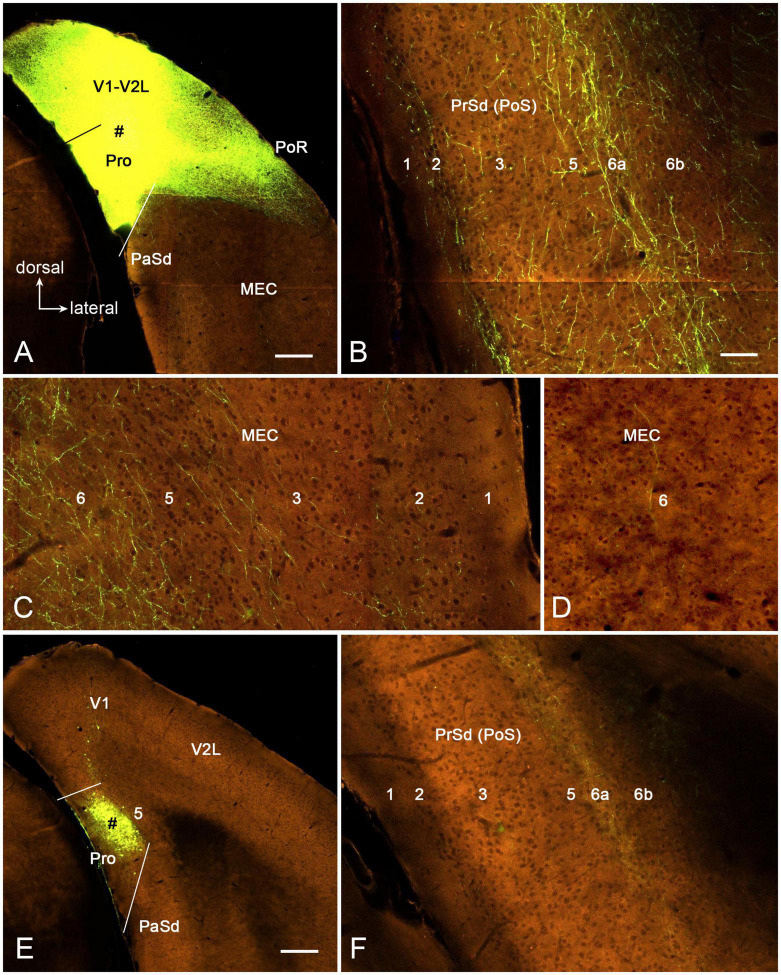
Axon terminal labeling following anterograde viral tracer injections in the prostriata of two wild-type mice. All panels are from coronal sections. **(A–C)** One large injection (# in **A**) involved in all layers of both prostriata and overlying visual cortex results in labeled axon terminals in PrSd (**B**, mainly in layers 2, 5, and 6a) and medial entorhinal cortex (MEC) (**C**, mainly in layer 6), respectively. **(D–F)** Another small injection (# in **E**) restricted in layers 2–3 of the prostriata results in labeled axon terminals in PrSd (**F**, mainly in layer 6a) with much less labeling in MEC (**D**, layer 6). Bars: 280 μm **(A)** for **(A,E)**; 70 μm **(B)** for **(B–D,F)**.

To further confirm above findings and reveal layer-specific efferent projections of area prostriata, the connectivity dataset of both wild-type and Cre-line mice from Allen Institute^1^ were searched; and the cases with injections of the anterograde viral tracers in mouse prostriata were examined. Two wild-type cases with injections (one large and the other small) in the prostriata are available for analysis. The large injection is involved in both the prostriata (all layers) and overlying visual cortex (all layers) (Figure 8A) and results in terminal labeling in all the target regions of the prostriata described above for rat (e.g., LP-Pul, VLG, and PTN) as well as other regions such as the DLG, SC, and nearby visual cortices, which are probably the target regions of the visual cortex (V1 and V2L). Figures 8B,C show the distribution of labeled axon terminals in the PrSd (mainly in layers 2, 5, and 6a) and MEC (mainly in layer 6), respectively. Contralaterally, labeled terminals are mainly found in layers 2–3 of the prostriata (see Chen et al., 2020), in addition to the typical callosal labeling patterns in the visual cortex (see Ding and Elberger, 2001). The small injection is restricted in layers 2–3 of the prostriata (Figure 8E) and mainly produces some labeled terminals in layer 6a of PrSd (Figure 8F) with much less labeling in layer 6 of the MEC (Figure 8D). Contralaterally, sparsely labeled terminals are seen in layers 2–3 of the prostriata (not shown).

**FIGURE 9:**
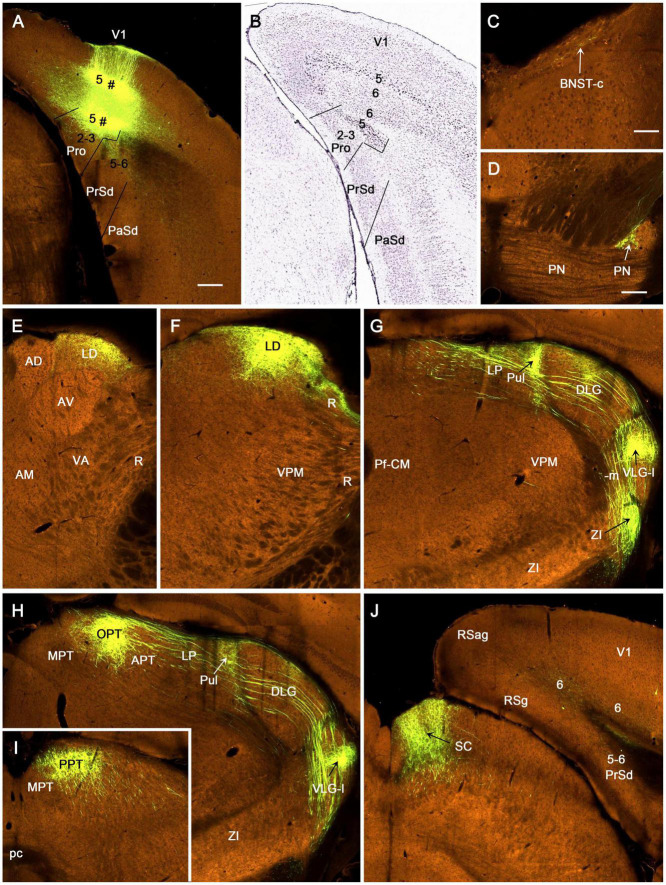
Axon terminal labeling following an anterograde viral tracer injection in prostriata and V1 of a *Npr3*-IRES2-Cre mouse. All panels are from coronal sections. **(A)** The injection site (marked by #) is involved in layer 5 of both prostriata and overlying V1. **(B)** One *in situ* hybridization (ISH)-stained section showing *Npr3*-Cre expression mostly in layer 5 of the prostriata and V1. **(C–J)** Distribution of labeled axon terminals. Locations of some terminal labeling are indicated by the arrows. Relatively strongly labeled terminals are detected in layers 5–6 of the PrSd **(A)**, PN **(D)**, LD **(E,F)**, dorsal part of reticular thalamic nucleus **(F)**, LP-Pul **(G,H)**, lateral part of the VLG (VLG-l; **G,H**), lateral part of zona incerta **(G)**, OPT and PPT of the PTN **(H,I)**, and medial part of the SC **(J)**. Weak terminal labeling is detected in the caudal part of the bed nucleus of stria terminalis (BNST-c in **C**), DLG **(G,H)**, and layer 6 of RSg, RSag, and visual cortices **(J)**. Bars: 280 μm **(A)** for **(A,B)**; 0 μm **(C)**; 200 μm **(D)** for **(D–J)**.

**FIGURE 10:**
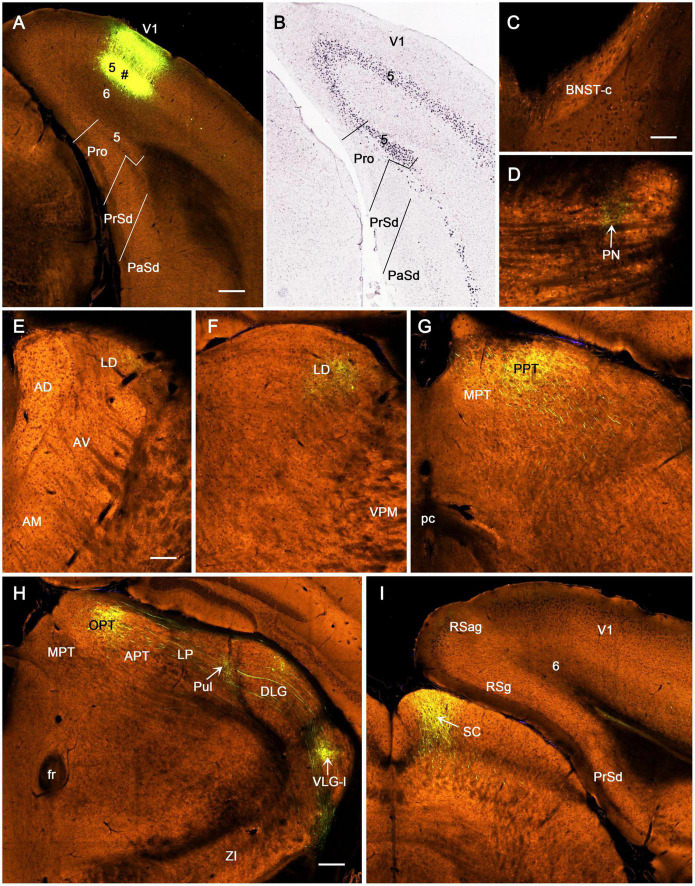
Axon terminal labeling following an anterograde viral tracer injection in V1 of an *Etv1*-CreERT2 mouse. All panels are from coronal sections. **(A)** The injection site (marked by #) is restricted in layer 5 of V1 without involvement in the prostriata. **(B)** One *in situ* hybridization (ISH)-stained section showing *Etv1*-Cre expression mostly in layer 5 of the prostriata and V1. **(C–I)** Distribution of labeled axon terminals. The labeled terminals are seen in PN **(D)**, LD **(E,F)**, OPT and PPT of the PTN **(G,H)**, Pul **(H)**, lateral part of the VLG (VLG-l in **H**), and medial part of the SC **(I)**. Note that **(G)** is caudal to **(H)**, which is at the similar level of the **(H)** in Figure 9. Note the absence of terminal labeling in the BNST-c **(C)**, lateral part of zona incerta **(H)**, and layer 6 of RSg and RSag **(I)**. Bars: 280 μm **(A)** for **(A,B)**; 70 μm **(C)** for **(C,D)**; 140 μm **(E)** for **(E–G)**; 200 μm **(H)** for **(H,I)**.

We have also analyzed one *Npr3*-IRES2-Cre case with an injection involved in layer 5 of the prostriata and overlying V1 (Figure 9A). In this mouse, *Npr3-*Cre expression is observed mostly in layer 5 of the prostriata and V1 (Figure 9B). This injection results in strong terminal labeling in the PN (Figure 9D), LD (Figures 9E,F), dorsal part of reticular thalamic nucleus (R; Figure 9F), LP-Pul (Figures 9G,H), lateral part of VLG (VLG-l; Figures 9G,H), lateral part of zona incerta (ZI; Figure 9G), OPT and PPT of PTN (Figures 9H,I), and medial part of SC (Figure 9J). Weak terminal labeling is seen in the caudal part of the bed nucleus of stria terminalis (BNST-c; Figure 9C), DLG (Figure 9G), and layer 6 of RSg, RSag, and visual cortices (Figure 9J). Interestingly, after an injection is restricted to layer 5 of V1 (Figure 10A) in an *Etv*1-CreERT2 mouse (Cre expression in layer 5; Figure 10B), the injection also results in clearly labeled terminals in most of the above-mentioned regions, although the labeling is relatively weaker (Figures 10D–I). However, no labeled terminals are detected in the BNST-c (Figure 10C) and layer 6 of RSg and RSag (Figure 10I). These findings suggest that layer 5 of the prostriata and V1 has more common than different output targets. Since injections restricted in rat prostriata produce no terminal labeling in medial SC (e.g., Figure 4F), the labeled terminals in the SC (Figure 9J) of the *Npr3*-Cre case probably originate from layer 5 of V1 (see Figure 10I).

### Direct Projections From Dorsal Lateral Geniculate Nucleus to Prostriata in Rat and Mouse

**FIGURE 11:**
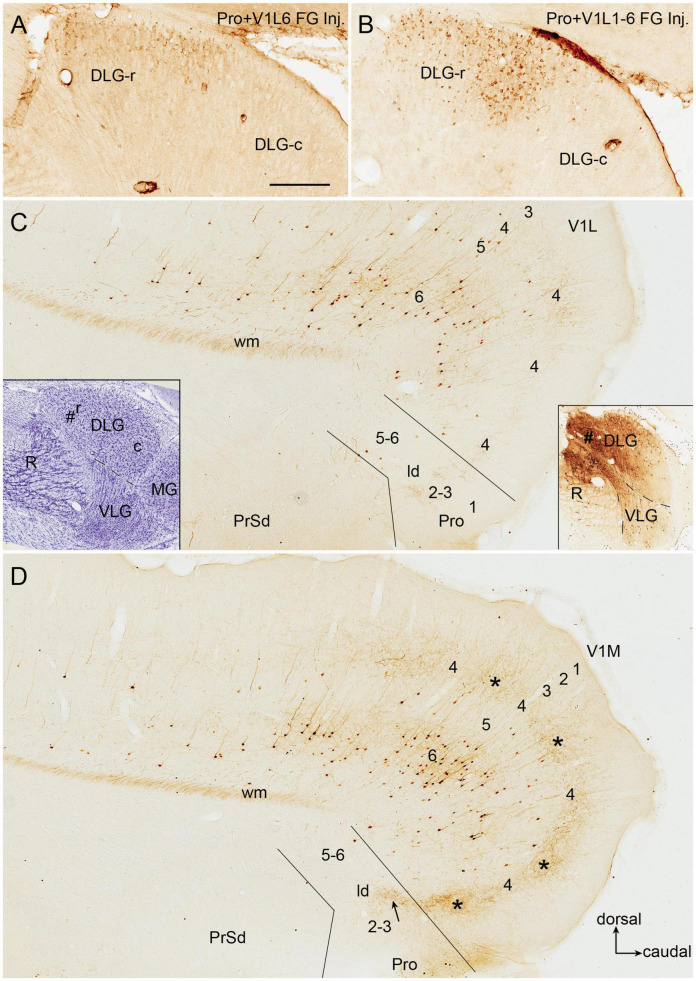
DLG-r projections to area prostriata and V1 in rat. All panels are from sagittal sections. **(A,B)** Retrogradely labeled neurons in DLG-r following Fluoro-Gold (FG) injections in Pro + V1L6 **(A)** and Pro + V1 (all layers) **(B)**. Note that much more neurons ware observed in the DLG-r in **(B)**. **(C,D)** Labeled neuronal somata and axon terminals in V1 and prostriata after a biotinylated dextran amine (BDA) injection restricted in the DLG-r (# in the right insets in **C**; the left inset shows the locations of the DLG, VLG, R, and MG on a matched Nissl-stained sagittal section). Many retrogradely labeled neurons are observed in layer 6 of V1 with much fewer in layer 5 of V1 and no or few in the prostriata on both lateral **(C)** and medial **(D)** sagittal sections. In contrast, labeled axon terminals are mainly seen on the medial section **(D)**. The labeled terminals mainly distribute in layer 4 and deep layer 3 of V1 with a patchy organization (indicated by the stars in **D**) as well as in dorso-caudal portion of lamina dissecans (indicated by the arrow in **D**).

Since the retrograde tracers injected in rat prostriata and Pro + V1L6 have resulted in some labeled neurons in the DLG-r (Figures 2I, 3E, 4E) and the injections in Pro + V1 (all layer) produce much more labeled neurons in the DLG-r (e.g., Figures 11A,B), we have tried to inject and restrict BDA into rat DLG-r to clarify whether the DLG-r projects to the prostriata and/or V1. As shown in Figures 11C,D, following a BDA injection in the DLG-r (# in the right inset of Figure 11C; the left inset showing the locations of the DLG and VLG on a matched Nissl-stained section), sparsely and densely labeled axon terminals are observed in the lateral (V1L; Figure 11C) and medial V1 (V1M; Figure 11D), respectively. The labeled terminals are clearly detected in layer 4 and deep layer 3 of V1 but not in layers 5–6, and these terminals tend to distribute in patches (indicated by the stars in Figure 11D). In the prostriata, sparse and moderate terminal labeling is seen in deep layer 3 and the dorsal part of ld (indicated by the arrow in Figure 11D), respectively, with no or few in layers 5 and 6 (Figures 11C,D). Finally, it worth mentioning that a great number of retrogradely labeled neurons are also seen in layer 6 of V1 with much fewer in layer 5 and few in the prostriata (Figures 11C,D), suggesting that layer 6 of V1 rather than the prostriata heavily innervates the DLG.

**FIGURE 12:**
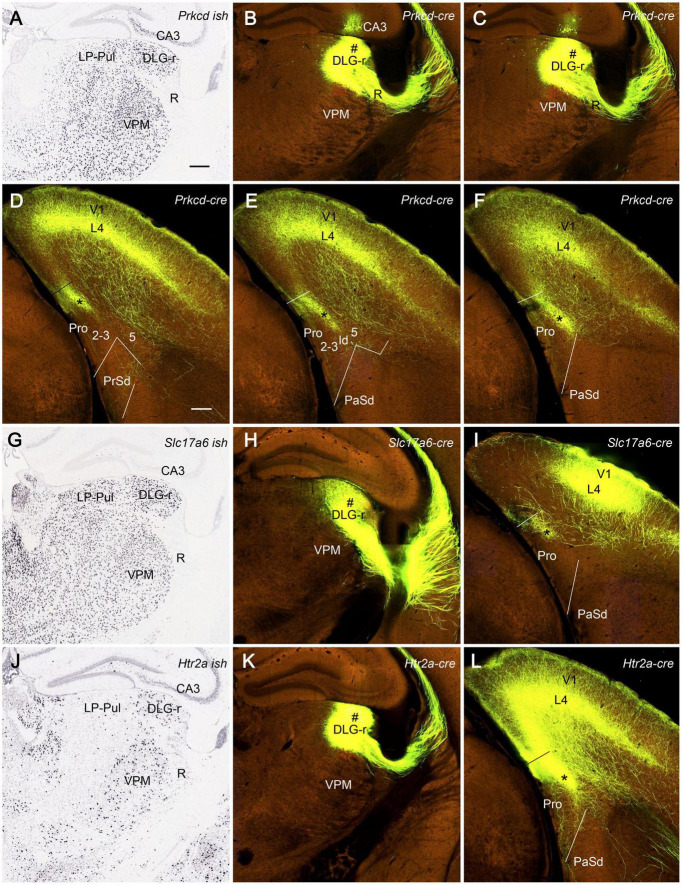
Efferent projections from DLG to prostriata in mouse. All panels are from coronal sections. **(A)** Expression of *Prkcd*-Cre in the thalamus of a Cre-line mouse. **(B,C)** One anterograde viral tracer injection (#) is involved in the DLG-r as well as a small portion of LP-Pul and ventral posteromedial thalamic nucleus (VPM) of a *Prkcd*-Cre mouse. **(D–F)** Strongly labeled axon terminals are detected in layer 3 and lamina dissecans (marked by the stars) of the dorsal prostriata as well as in layer 4 and deep layer 3 of V1 as shown on sequential coronal sections from rostral **(D)** to caudal **(F)** levels. **(G)** *Slc17a6*-Cre expression in the thalamus. **(H,I)** Another injection mainly located in the intermediate part of DLG with partial involvement in the DLG-r (# in **H**) of a *Slc17a6*-Cre mouse produces terminal labeling in the dorsal prostriata only at the caudal level (indicated by the star in **I**). **(J)** *Htr2a*-Cre expression in the DLG-r of the thalamus with much less expression in nearby region. **(K,L)** The third injection (#) mainly involved in the DLG-r **(K)** of a *Htr2a*-Cre mouse results in strongly labeled axon terminals in layer 4 and deep layer 3 of V1, as well as in layer 3 and lamina dissecans (indicated by the star in **L**) of the dorsal prostriata along rostro-caudal levels (**L** showing only the caudal level image). Bars: 285 μm **(A)** for **(A–C,G,H,J,K)**; 200 μm **(D)** for **(D–F,I,L)**.

We have also searched the Allen Institute dataset for the cases with anterograde viral tracer injections in the DLG-r and found four of these cases (e.g., Figure 12). Two cases are from *Prkcd*-GluCla-CFP-IRES-Cre mice, in which *Prkcd*-Cre is expressed in the DLG, LP-Pul, and ventral posteromedial thalamic nucleus (VPM) (Figure 12A), and the injections are involved in these three regions (e.g., Figures 12B,C). Strongly labeled axon terminals exist in layer 4 and deep layer 3 of V1 as well as in layer 3 and ld of the dorsal prostriata along rostro-caudal levels (Figures 12D–F), in addition to terminal labeling in primary somatosensory cortex and other regions (not shown). One injection in *Slc17a6*-IRES-Cre mouse (Cre expression in the DLG, LP-Pul, and VPM; Figure 12G) is located at the more caudal level and mainly involved in the intermediate part of the DLG with partial involvement in the DLG-r (Figure 12H). This injection leads to terminal labeling in the dorsal prostriata only at the caudal level (Figure 12I). Another injection is placed in one *Htr2A*-Cre_KM207 mouse (Cre expression mainly in the DLG and VPM; Figure 12J), and the effective injection is located mostly in the DLG-r (Figure 12K) and partially in the nearby VPM. This injection produces strongly labeled axon terminals in layer 4 and deep layer 3 of V1 as well as in layer 3 and ld of the dorsal prostriata along rostro-caudal levels (similar to levels D–F; Figure 12L showing the labeling at the caudal level). Since no terminal labeling was found in the prostriata when the tracers were restricted in the VPM and other part of the DLG (not shown), the labeled axon terminals observed in the prostriata in above cases probably originate from the DLG-r.

## Discussion

**FIGURE 13:**
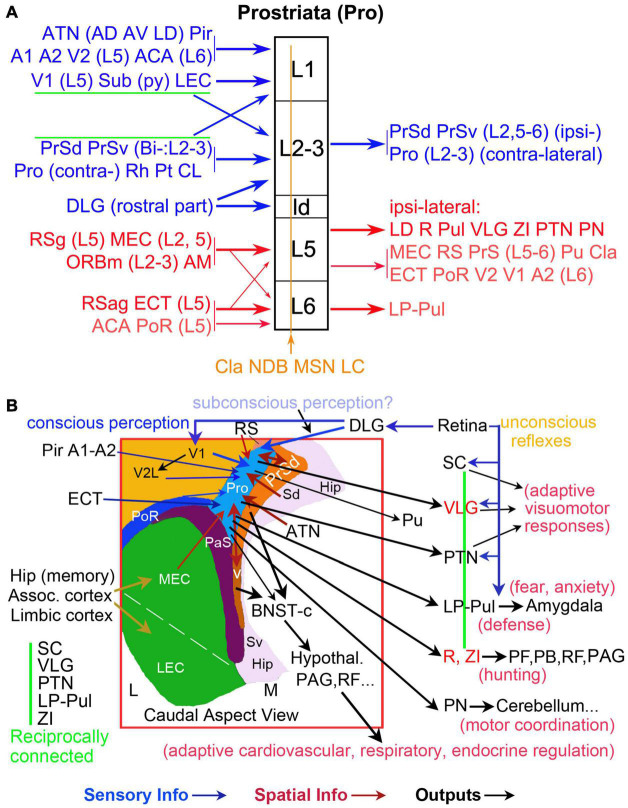
Summary of afferent and efferent projections and wider networks of the prostriata in rodent. **(A)** Laminar organization of prostriata connectivity. The layers of prostriata (in black) are shown in the middle column. Main afferent and efferent projections are displayed at left and right columns, respectively. Connectivity of the superficial and deep layers of prostriata are, respectively, coded blue and red. Diffuse afferents to all layers of prostriata are coded brown. Thicker and thinner arrows indicate stronger and weaker projections, respectively. Note that layers 2–3 of the prostriata mainly project to PrS and contralateral counterpart (see Chen et al., 2020) and the subcortical outputs of the prostriata mainly originate from layer 5. **(B)** Topography and wider anatomical networks of prostriata with correlated functions. A schematic map of the region containing prostriata and adjoining areas is shown in the boxed region, which is a caudal aspect view of the left hemisphere. Major sensory inputs (blue arrows), spatial information inputs (red-brown arrows), and outputs (black arrows) are indicated. The vertical green line indicates the five reciprocally connected structures as common effectors of many visuomotor behaviors. The VLG, R, and ZI are coded in red for the feature of mostly GABAergic inhibitory neurons. In general, the direct retinal projections to SC, VLG, PTN, and LP-Pul could be the pathways underlying unconscious reflexes, while the retina–DLG–V1–V2 pathways could be required for conscious perception. The retina–DLG–prostriata pathways likely contribute to subconscious perception and blindsight.

The present and our recent studies (Ding, 2013; Ding et al., 2020; Hu et al., 2020; Lu et al., 2020) demonstrate that the prostriata in rat and mouse receives inputs from many cortical and subcortical regions. The cortical inputs originate mainly from primary sensory (V1, A1, and Pir), association sensory (V2L and A2), polymodal association (ORBm, ECT, and PoR), and limbic (ACA, RS, MEC, LEC, Sub, and PrS) cortices. The subcortical inputs derive mainly from ATN (AD, AV, AM, and LD), some intralaminar or midline thalamic nuclei (Pt, CL, and Rh), NDB, and Cla (Figure 13A). One additional and important finding of the present study is the direct projections from the DLG-r to the prostriata in rat and mouse, supporting a recent finding in the human brain based on tractography (Kurzawski et al., 2020). As for the outputs, we find that rodent prostriata mainly targets the PrS, LD, LP-Pul, VLG, PTN, R, ZI, and PN (Figure 13A) as well as the contralateral prostriata (Chen et al., 2020). Weaker efferent projections are observed in many of its afferent source regions (e.g., MEC, RS, ECT, PoR, V2, V1, A2, ORBm, and Cla).

### Multimodal Hierarchical Inputs Converge in Area Prostriata

In our recent studies, we have discussed the convergence of the multimodal and bilateral inputs to the prostriata (Chen et al., 2020; Hu et al., 2020). Among these inputs, many appear to be modulatory or feedback projections since they mainly target layer 1 or layers 5–6 of the prostriata. These inputs include those from primary and secondary olfactory, auditory, and visual cortices as well as those from the ATN (AD, AV, AM, and LD), Sub, RS, and MEC, which are important for spatial processing and navigation. Projections from ATN to the prostriata were also noted in the tree shrew (Conrad and Stumpf, 1975). The inputs to the prostriata from the ipsilateral PrS and DLG-r as well as contralateral counterpart are very strong and appear to be feedforward projections since they innervate prostriata’s layer 3 and the dorsal part of lamina dissecans (ld), which adjoins V1 and contains some granular cells corresponding to weak layer 4 in monkey and human prostriata (Ding et al., 2003, 2016). In general, the projections from lower to higher regions of the hierarchy are termed feedforward projections, which mainly target middle layers in the cerebral cortex (layer 4 and deep layer 3). Those projections from higher to lower regions of the hierarchy are referred as feedback projections, which mainly innervate layers 1 and 5–6 in the cortex (Rockland, 1997, 2019; Mesulam, 1998; Markov et al., 2014). The convergence in the prostriata of multiple primary, secondary, association sensory information with limbic inputs suggests that the prostriata is an important integration hub for different but related information about the objects in far peripheral visual field (Figure 13B).

### Neural Substrates for Rapid Processing of Fast-Moving Stimuli in Peripheral Visual Field

The prostriata in monkeys and humans was reported to play important roles in fast and coarse analysis of moving and unexpected stimuli in far peripheral visual field (Yu et al., 2012; Mikellidou et al., 2017; Tamietto and Leopold, 2018). Monitoring peripheral visual filed is critical for detection of sudden changes in environmental conditions such as unexpected looming threat and for self-motion stabilization and head and body orientation (Palmer and Rosa, 2006). However, neural circuits underlying the fast processing in the prostriata are basically unknown. Recently, we have reported the existence of direct projections from V1 to the prostriata in rat and mouse (Lu et al., 2020). Since the prostriata belongs to the limbic cortex, this pathway would provide a much shorter relay from V1 to the limbic cortex than the typical hierarchical pathway from V1 to V2 to polysensory cortex and limbic structures (see Rockland, 2012). Moreover, the mediocaudal V1 region, which represents far peripheral visual field, has been found to send stronger projections to the prostriata than the laterorostral V1 region, which represents central visual field (Lu et al., 2020). This finding is consistent with the specialized role of the prostriata in monitoring fast-moving stimuli in far peripheral visual field.

Interestingly, another recent study of human brains has suggested the existence of direct projections from the DLG to the prostriata (Kurzawski et al., 2020). In the present study, we have confirmed the presence of these direct projections in rat and mouse using both retrograde and anterograde tract tracing methods. Moreover, we find that these projections originate only from the DLG-r, which appear to receive direct inputs from the part of retina representing far peripheral visual field (Piscopo et al., 2013). Since no projections were reported directly from the retina to the prostriata, the retina–DLGr–prostriata projections revealed in this study would represent the shortest visual pathway from the retina to the limbic cortex (prostriata) and serve as a fast neural circuit for rapid analysis of fast-moving objects in far peripheral visual field (Figure 13B).

In general, lateral geniculate body/nucleus (LGN) in rodent, cat, tree threw, and some others includes both the DLG and VLG (e.g., Agarwala et al., 1992; Nakamura and Itoh, 2004; Paxinos and Watson, 2013; Wang et al., 2020), although the DLG was sometimes treated as LGN in literature due to its much larger size in primate over rodent (e.g., Kurzawski et al., 2020). The equivalent of the VLG in humans and monkeys (usually very small in size) is often termed pregeniculate nucleus (PG) mainly because it is located rostrodorsal to the DLG rather than ventral to the DLG in adult brain (e.g., Paxinos et al., 2012; Ding et al., 2016). Interestingly, the PG is located ventromedial to the DLG during early prenatal development of human brain (Ding et al., 2021). To our knowledge, it is currently not clear which part of the DLG in non-rodent corresponds to the DLG-r in rodent.

Finally, it should be mentioned that many previous studies supported some subcortical pathways such as the retina–SC–pulvinar pathway as the neural circuits for fast and unconscious processing of visual information and the blindsight since this pathway bypasses the V1 (McFadyen, 2019; Fox et al., 2020; Isa et al., 2021). However, this pathway does not appear to process fast-moving visual stimuli, although the neurons in the SC, for example, are involved in the detection of salient visual stimuli and the processing of spatial and temporal frequency, contrast, and orientation (White et al., 2017; Chen and Hafed, 2018; Chen et al., 2018). Therefore, it is likely that the processing of fast-moving objects in far peripheral visual field occurs in the retina–DLGr–prostriata pathway, which also bypasses V1 and could enable blindsight following V1 damage and fast adaptive visuomotor responses via direct projections from the prostriata to the PTN, VLG, Pul, and ZI (Figure 13B).

### Output Pathways of Area Prostriata

In literature, the prostriata in monkeys was reported to project to V1 (Sousa et al., 1991), middle temporal visual area (Rosa et al., 1993; Palmer and Rosa, 2006), orbitofrontal cortex (Barbas, 1993; Cavada et al., 2000), rostral cingulate motor cortex (Morecraft et al., 2000), auditory association cortex (Falchier et al., 2010), dorsal prefrontal cortex (area 8b) (Reser et al., 2013; Bakola et al., 2021), and frontal pole (Burman et al., 2011). The projections from the prostriata to V1, orbitofrontal, and auditory association cortex have been confirmed in rodents (Lu et al., 2020 and this study); and these projections are relatively weak (this study). Since the equivalents of the monkey middle temporal area and rostral cingulate motor cortex have not been identified in rodent, it is not clear if these two regions (if existence) receive projections from rodent prostriata. The present study reveals that the major target structures of rodent prostriata are the PTN, VLG, LP-Pul, LD, R, ZI, PN, and PrS (including postsubiculum). Many of these structures are important for visuomotor behaviors (see below), while PrS is critical to head direction and orientation.

The present study demonstrates that the PTN receives relatively strong projections from the prostriata. These projections originate from layer 5 of the prostriata and mainly terminate in the OPT and PPT of PTN. The OPT, for instance, plays an important role in pupillary light reflex pathway, in which it receives luminance information from intrinsically photosensitive retinal ganglion cells and sends direct projections to preganglionic motoneurons in the Edinger–Westphal nucleus (Burde and Williams, 1989; Gamlin, 2006). The VLG (lateral part) receives dense innervation from the retina (Hickey and Spear, 1976; Monavarfeshani et al., 2017) and gives rise to intensive projections to many subcortical structures related to visuomotor functions such as the LP-Pul, PTN, and SC (Kolmac et al., 2000; Moore et al., 2000; Monavarfeshani et al., 2017; Ciftcioglu et al., 2020) and to the BNST-c (our unpublished data), which receives dense inputs from the PrS-PoS (Ding, 2013). Neurons in the VLG sensitive to big bright stimuli contact remote structures via long-range inhibitory GABAergic synapses and enable rapid movement by releasing motor targets like the deep SC from suppression (Monavarfeshani et al., 2017; Ciftcioglu et al., 2020; Isa et al., 2021). The LP-Pul, which receives direct projections from the prostriata, were reported to be important in visual attention and orientation as well as fear processing via its direct connections with SC and amygdala (Arend et al., 2008; Wei et al., 2015; McFadyen, 2019). The ZI, containing mostly GABAergic neurons, is important for hunting and motivation via its projections to the periaqueductal gray (PAG; Zhao et al., 2019) and other structures such as the parafascicular nucleus (PF), parabrachial nucleus (PB), brainstem reticular formation (RF), SC, PTN, and VLG (Figure 13B). Generally, SC, VLG, PTN, LP-Pul, and ZI are reciprocally connected with each other, forming common effectors for visuomotor behaviors (Figure 13B). Since the location and extent of the prostriata in human brain have been detailed in sequential histological and MRI slices (Ding et al., 2016), it would be interesting to see in the future how the prostriata, pulvinar, and amygdala respond to fear stimuli in human brains.

In summary, the present study and our recent studies suggest that area prostriata probably plays important roles in the fast integration of information about fast-moving objects and related sounds or smells in peripheral environment (Chen et al., 2020; Hu et al., 2020; Lu et al., 2020). The present study also suggests that the prostriata is positioned to quickly initiate and modulate adaptive visuomotor responses via its direct efferent projections to the subcortical effectors such as the PTN, VLG, LP-Pul, LD, R, ZI, and PN, particularly in response to unexpected looming threats. Finally, the direct projections from the DLG-r to the prostriata could also contribute to blindsight after V1 lesion since this pathway bypasses V1 (Figure 13B).

## Data Availability Statement

The original contributions presented in the study are included in the article/supplementary material, further inquiries can be directed to the corresponding author/s.

## Ethics Statement

The animal study was reviewed and approved by the Institutional Animal Care and Use Committee, Guangzhou Medical University.

## Author Contributions

S-LD: experimental design. C-HC, J-MH, S-YZ, and X-JX: investigation. C-HC and S-LD: data analysis and writing. S-LD and S-QC: supervision. All authors contributed to the article and approved the submitted version.

## Conflict of Interest

The authors declare that the research was conducted in the absence of any commercial or financial relationships that could be construed as a potential conflict of interest.

## Publisher’s Note

All claims expressed in this article are solely those of the authors and do not necessarily represent those of their affiliated organizations, or those of the publisher, the editors and the reviewers. Any product that may be evaluated in this article, or claim that may be made by its manufacturer, is not guaranteed or endorsed by the publisher.

## Footnotes

1
https://connectivity.brain-map.org
